# LUX: Smart Mirror with Sentiment Analysis for Mental Comfort

**DOI:** 10.3390/s21093092

**Published:** 2021-04-29

**Authors:** Hyona Yu, Jihyun Bae, Jiyeon Choi, Hyungseok Kim

**Affiliations:** School of Intelligent Mechatronics Engineering, Sejong University, Seoul 05006, Korea; gysk97@sju.ac.kr (H.Y.); jihyun4133@sejong.ac.kr (J.B.); choiwldus@sju.ac.kr (J.C.)

**Keywords:** sentiment analysis, smart mirror, NLP, deep learning, raspberry Pi, affective computing

## Abstract

As COVID-19 solidifies its presence in everyday life, the interest in mental health is growing, resulting in the necessity of sentiment analysis. A smart mirror is suitable for encouraging mental comfort due to its approachability and scalability as an in-home AI device. From the aspect of natural language processing (NLP), sentiment analysis for Korean lacks an emotion dataset regarding everyday conversation. Its significant differences from English in terms of language structure make implementation challenging. The proposed smart mirror LUX provides Korean text sentiment analysis with the deep learning model, which examines GRU, LSTM, CNN, Bi-LSTM, and Bi-GRU networks. There are four emotional labels: anger, sadness, neutral, and happiness. For each emotion, there are three possible interactive responses: reciting wise sayings, playing music, and sympathizing. The implemented smart mirror also includes more-typical functions, such as a wake-up prompt, a weather reporting function, a calendar, a news reporting function, and a clock.

## 1. Introduction

Sentiment analysis is a technology that perceives the pattern of human emotion and is commonly used as a supplement in treating mental issues. It has gained steady attention, specifically in dialog systems. With rapid improvements in artificial intelligence (AI), better sentiment analysis has become possible; sentiment analysis can now use data based on natural language, human speech intonation, vision-based emotion detection, or human gesture [1]. Since several AI systems that use sentiment analysis are based on user speech, the primary data for classifying sentiment is natural language text [2]. Classifying emotion via text has improved company responses to online reviews and the targeting of news articles on social network sites. Lexicons and the tagging of sentiment speech are used widely for sentiment analysis [3]. The application of sentimental analysis can also be helpful to patients coping with medical conditions [4]. Particularly relevant to the present day, as the ongoing COVID-19 pandemic is causing widespread depression and anxiety in human beings, sentiment analysis is a viable way to detect and prevent their symptoms.

However, despite the existence of in-home AI devices such as smart doorbells, AI speakers, and smart mirrors, sentiment analysis has yet to be popularized. According to a previous study, a user uses an AI speaker for the following reasons: 82% for the sensation of company, 61% for increased confidence, and 77% for general happiness [5]. For instance, Amazon’s Alexa was favored with a score of 4.32 out of 5, and the most frequent interaction type, at 79%, was found to be entertainment [6]. These studies imply that users find value in in-home AI devices as emotional support, but this demand is not being actively met. Besides, the global smart mirror market scale is growing; the retail sales of 2020 add up to 27.7 trillion USD, increasing by 1.4 trillion USD compared to 2019 [7]. The growing market of smart mirrors and their extensibility from familiar hardware inspired this paper to suggest applying sentiment analysis to this particular AI device.

As an interactive interface with user sentiment in affective computing, users become familiar with smart mirrors [8]. Studies on smart mirrors have made continuous progress. A recent study on the smart mirror for personalized home use suggests integrating face recognition for authentication, using OpenCV and voice commands to interact with custom commands that have already been registered [9]. The smart mirror is widely seen as the conjugation of an ambient mirror [10]. A smart mirror, an evolving home appliance with a familiar surface as an interface, also establishes itself as a device that focuses on human convenience. A previous study [11] has suggested that this framework can allow interactive user communication when implemented into a house automation device, displaying a compliment, the weather, the current time, a calendar, and the news. FitMirror [12] helps users wake up and encourages them to exercise with the supervising mirror. There is a study on a voice-controlled smart mirror for voice-based command [13]. It also takes advantage of the user-friendly architecture by providing end-to-end security. Their work focuses on the smart mirror display, which is around the entire home using a smart projector. Another study investigated human posture detection for health care purposes [14] by applying a posture analysis algorithm. The analysis results showed that this algorithm enhanced upright posture at a considerable rate. These studies demonstrate the rationale of implementing sentiment analysis in smart mirrors.

Recent sentiment analysis studies have investigated voice intonation, facial emotion detection, and text-based algorithms that use deep learning algorithms to solve NLP tasks. The accomplishment of Speech-to-Text (STT) provides insight into receiving useful messages, such as social and psychological intonation signals [15]. The current NLP study of sentiment analysis also tries to recognize binary classifications, such as whether a message is positive or negative, to capturing various emotions such as love, joy, anger, sadness, fear, and surprise [16].

This paper proposes an evolved smart mirror that analyzes user sentiment and manages sentiment feedback. The proposed smart mirror with sentimental analysis in the Korean language is named LUX (an abbreviation of language user experience). For sentiment analysis, training datasets are required to train the model. LUX uses two datasets: a short conversation dataset and the Instagram-crawling dataset. The AI Hub dataset which includes online reviews is organized into four categories: sadness, anger, neutral, and happiness [17]. The second dataset, the Instagram-crawling dataset, is applied for reflecting conversations in everyday life. This dataset is organized into intensively three categories: sadness, anger, and happiness. LUX uses Text-to-Speech and Speech-to-Text (TTS-STT) API from the Google cloud platform (GCP) and Google’s virtual machine (VM) for cloud computing. LUX returns different responses to each sentiment with the purpose of making the user feel better. These responses include reciting wise sayings, empathizing, and playing MP3 music as music therapy. These responses are expected to help control the user’s emotional agitation. The main contributions of the paper are as follows:

- Using a deep learning model to build a smart mirror with user sentiment analysis, and,
- Providing responses that encourage specific emotions in users.

The paper is organized as follows. Section 2 discusses related works on sentiment analysis tasks. Section 3 describes the sentiment analysis model and preprocessing procedures, including the entire software model of LUX. Section 4 introduces an interaction between the speaker, microphone modules, and LUX architecture. Section 5 reports the evaluation results of the sentiment analysis implemented in LUX. Section 6 concludes this paper.

## 2. Related Works

Due to the remarkable performance of deep learning, neural networks have played a significant role in language models. In NLP for sentiment analysis, the text or sentence data has a characteristic of sequence. The data are suitable for comparing and classifying emotions through RNN for modeling sequential data. Long short-term memory (LSTM), a type of RNN, computes the input memory with a nonlinear function. It has cell states and gates, such as an output gate, an input gate, and a forget gate, to handle the memory cell extracting the feature and avoid the vanishing gradient problem and long-term dependency [18].

Studies on Korean sentiment analysis are given below. A study of building a Korean sentiment lexicon constructed sentiment word dictionary using collective intelligence [19]. It provides word tagging with positive, negative, and neutral categories, voted by whether it is positive or negative by collective intelligence participants. The dictionary works as a sentiment analysis tool and expresses emotion by independent status, not considering contexts. Another study examines the sentiment of Cyworld, a social networking site (SNS), regarding the personal profile of politicians [20]. They selected 200 comments of politicians and classified them into three labels, positive, negative, and irrelevant. A study of Korean linguistic features for sentiment analysis [21] uses a support vector machine (SVM) to evaluate model affixes from news, editorial, and subtopic news articles. Another study on Korean linguistic features proposed a corpus-based approach by constructing the corpus annotated with 25 emotion classes [22]. They classified constructed corpus of emotion classes to positive and negative and evaluated it by using SVM. The study of sentiment lexicon embedding [23] suggests joint encoding morphemes, part of speech (POS) tags, and embedding space with meaningful lexical morphemes. It uses attention-based LSTM networks for classification, and the proposed embedding was compared to other embedding methods and showed outperformance. A study on machine learning-based Korean Twitter emotion [24] presented the automatic build of fine-grained emotion lexicon sets. This method improved classification performance using SVM. A Korean review sentiment analysis study [25] implemented morpheme vectors with CNN to classify positive and negative. They evaluated their preprocessing methods and concluded that the deep learning model CNN shows the highest accuracy.

There are recent works of sentiment analysis in other languages. The Persian sentiment analysis framework’s study proposed a hybrid framework adopting word polarity detection [26]. It integrates deep neural networks (DNN), Persian dependency-based rules, convolution neural networks (CNN), and LSTM. The dataset was about product and hotel reviews, and it has two labels, positive and negative, which is different from ours, a four labels classifier. The best F1-score was 0.89 in the proposed hybrid classifier. Arabic sentiment analysis (ASA) was proposed by implementing deep learning with various preprocessing tasks [27]. It uses CNN and LSTM models with preprocessing methods such as tokenizing, stemming, normalization, and removing stop words. The dataset consisted of Tweets, with positive and negative classes. The result shows the highest evaluation performance when using CNN.

GRU has an advantageous architecture of RNN, similar to LSTM but with fewer weights to compute. One of the gates, the reset gate, forgets the past information and multiplies the activation. Candidate activation uses the result of the reset gate to calculate the candidate of current information. The update gate, which is a combination of the LSTM’s forget and input gates, decides the activation update rate. A previous study [28] empirically evaluated the GRU of three polyphonic music datasets and raw speech signal datasets, suggesting that GRU is capable of sequential data. It compared LSTM, GRU, and tanh units, and its result demonstrates that GRU-RNN outperforms and has faster CPU time progress, although, for LSTM and GRU units, it is hard to decide which one is better. The hierarchical multi-input and output bidirectional GRU (Bi-GRU) was proposed [29] for multi-label sentiment analysis of customer reviews in both lexical and semantic contexts. It uses a part of speech and word as input data. Although the dataset was a review of the product rather than conversations, the multi-label semantic word classifying task with the GRU model has demonstrated exemplary performance. As these previous studies have shown, GRU has the advantage of fast computation and shows good performance in sentiment analysis tasks.

## 3. Sentiment Analysis Model and Software of LUX

**Figure 1:**
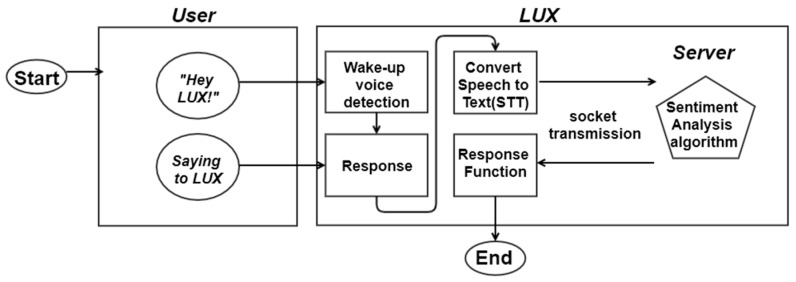
The proposed software framework of smart mirror LUX.

The proposed software framework of smart mirror LUX is shown in Figure 1. When the user calls LUX, the wake-up voice is detected. In the following step, LUX gives the response in Korean: “Shine with me, Yes I am here.” The next step involves the user speaking a simple sentence, which is converted to text through STT API. The text data are then transmitted using the socket method to the server, and the probability of a given emotion is calculated. The model inside the server is a soft ensemble model, including all tokenizing and preprocessing procedures. The next-to-last step is the sentiment analysis deep learning model. Finally, the response function calls forth one of the three responses. The five primary purposes are the wake-up voice model, training dataset, preprocessing of sentences, emotion detection model, and corresponding function.

### 3.1. Wake-Up Voice Model

**Figure 2:**
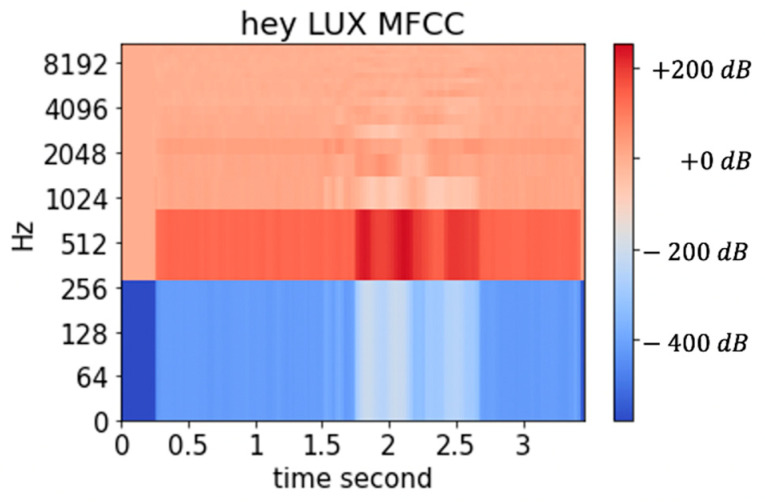
Example of “Hey LUX” MFCC.

The wake-up voice model of LUX involves the wake-up prompt “Hey LUX”. The thread buffer captures the voice data in real-time, and the preprocessing procedure converts the data list to Mel Frequency Cepstral Coefficient (MFCC). Figure 2 shows one of MFCC examples of “Hey Lux”. We compared the CNN, LSTM, and GRU networks using three labels: the preprocessed wake-up prompt, calm dummy, and noise dummy. The window stride is (160, 80) with 20 filters with sample rate 16,000, 13 coefficients, and Fast Fourier Transform (FFT) set by 512.

### 3.2. Training Dataset

A dataset for Korean sentiment analysis has recently been published by AI Hub established by the national information society agency (NIA). A combined dataset consisting of the AI Hub dataset and our Instagram crawling dataset is used. Within the emotion labels, four labels are selected to use in the smart mirror, such as anger, sadness, neutral, and joy. The numbers of sentences corresponding to each label are 5665, 5267, 4830, and 6037, respectively, a total of 80% of this data is for training, and the other set is for evaluation. This text dataset consists of online comments and SNS messages; therefore, it does not include a sufficient number of everyday conversation sentences. The everyday conversation dataset is needed to interact with users more appropriately.

The other one is an Instagram-crawling dataset, inspired by Twitter data’s sentiment analysis [30]. We crawled Instagram using hashtags in order to maintain maximum objectivity. As the neutral label is challenging to crawl due to its inherent vagueness, we rarely collected it and intensively crawled the remaining three labels, as shown in Table 1. There are 535, 808, 656, and 31 sentences for anger, sadness, happiness, and neutral, respectively, out of a total of 2030 sentences. The hashtags corresponding to “anger” were “I am angry” in Korean. The hashtags corresponding to “sadness” were “I am sick”, “Sigh”, and “I am exhausted” in Korean. The happiness keywords were “I am happy” and “This is a good day” in Korean, as well as the emoticon that indicates happiness. “ETC” means extra keywords to collect sentences. These data are selected only in Korean and exclude pictures.

### 3.3. Preprocessing

**Figure 3:**
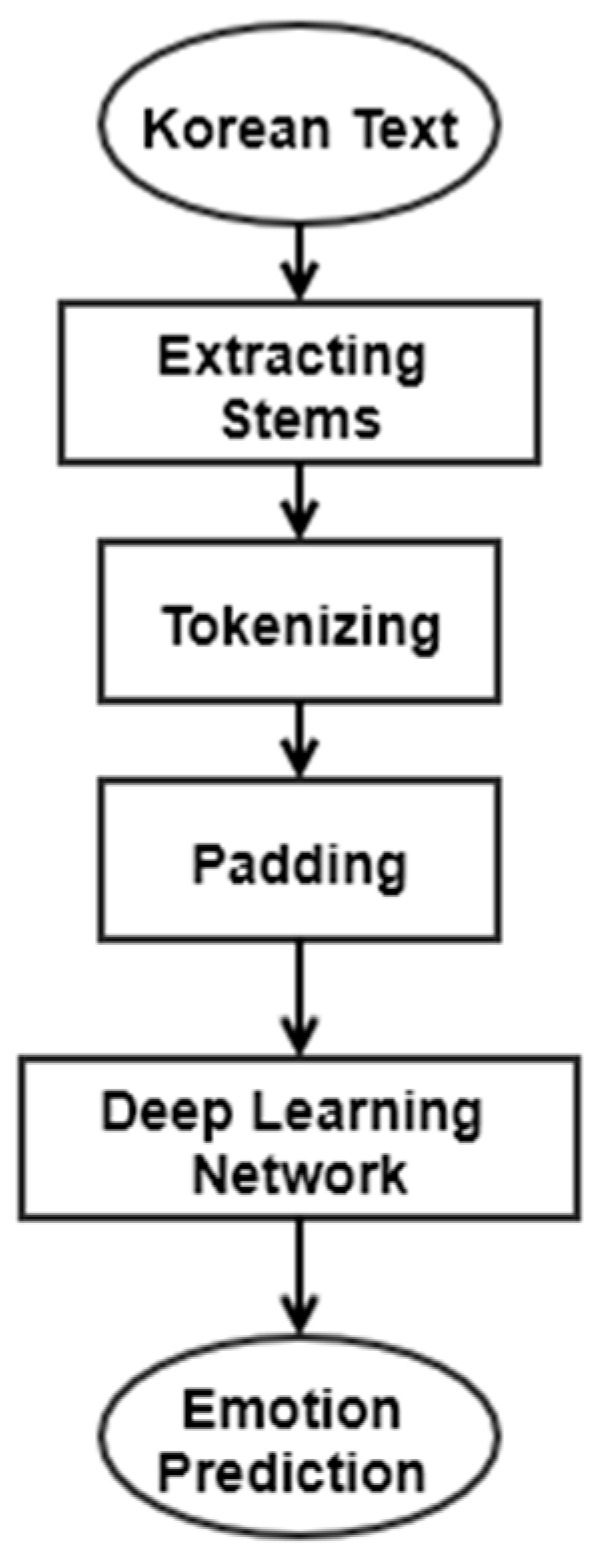
Structure of LUX sentiment analysis.

Before performing the sentiment analysis task with deep learning, several preprocessing steps such as STT and tokenizing are followed. First, the speech must be converted to text so that the NLP task will operate. The architecture of the preprocessing and machine learning modeling process is illustrated in Figure 3. It shows the procedure following the STT returning Korean text. First, the text is converted to stems, and a tokenizing process with padding is followed.

#### 3.3.1. Speech-to-Text

When the speaker module of LUX catches the audio signal, the voice is transformed into sentiment analysis. STT-TTS is also utilized to output proper responses that allow active user interaction.

#### 3.3.2. Tokenizing

Preprocessing specific to the Korean language is required before training. The Korean language does not allow given words to be divided using a spacing method, as any given word has two meanings: a stem and an affix. Therefore, the procedure of carrying out lemmatization before vectorizing a sentence plays an essential role in the preprocessing task. KoNLPy is an open-source package for preprocessing Korean words with Python, splitting the word into a stem and an affix [31]. We use OKT class which is the fastest inside the KoNLPy package, to preprocess Korean and divide texts into morphemes. After OKT returns the texts’ stems, the tokenizing task is followed using Keras tokenizer function by vectorizing stems and applying padding to deal with all vectors to equal length. In Keras tokenizing processing, a word’s maximum index is 20,542, and the output dimension is 50. The padding was set to have a vector length of 14, which contains 90% of the training dataset. However, if only the Instagram-crawling dataset would be used, the padding would have a vector length of 18 as the mean length of sentences. In short, the tokenizing process is:

- First, extracting stems via an OKT KoNLPy package,
- Second, using Keras Tokenizer for converting stems to vectors.

### 3.4. Sentiment Analysis Model

The paper experiments with several deep learning models, GRU, LSTM, CNN, Bi-LSTM, and Bi-GRU models, to perform the Korean sentiment analysis task to classify the emotional text. The programming language and libraries are Python, Tensorflow, Keras, and Pyaudio.

#### 3.4.1. GRU Network

GRU is similar to LSTM but lightweight compared to other NLP networks. First, the tokenized data are fed into the Embedding layer and then GRU with 128 neurons. The activation function is tanh since it is suitable for recurrent characteristic networks. A rate of 0.5 of the dropout layer prevents overfitting, and a dense output layer with a softmax is the next step. The output dimension is 4, which is the number of labels. The loss function is categorical cross-entropy loss to discriminate the labels adequately. The final optimizer is Adam applying the learning rate 1 × 10^−3^, with a 0.6 gradient clipping value to avoid gradient exploding. This network has 30 batch sizes in 15 epochs and appends early stopping if the validation loss does not grow down.

#### 3.4.2. LSTM Network

The following deep learning model is LSTM [23], which is specialized to handle sequential models. This algorithm has the same hyperparameters as those of GRU, embedding layer with vocabulary size and dense layer with 64 neurons and tanh activation function. After passing the dropout layer with a rate of 0.5, the output layer with the softmax function ends with the Adam optimizer, the way GRU does. The training has 15 epochs with the same batch size of 30 and early stopping callbacks as the GRU network.

#### 3.4.3. CNN Network

The deep learning method CNN as the binary classifier for sentiment analysis is used in [25] and it is adopted to compare with others. CNN network has 128 neurons and a kernel size of 3. Unlike other networks, rectified linear unit (ReLU) activation function would be used instead of tanh. The next step of the convolution network is the global max-pooling layer. After the flatten layer, a dense layer with four neurons with softmax activation function follows to extract output predictions. Training epochs, dropout rate, and batch size are the same as others.

#### 3.4.4. Bi-LSTM Network and Bi-GRU Network

For the comparison, Bidirectional-LSTM (Bi-LSTM) and Bi-GRU models are implemented. The two models have the same architecture, 128 neurons with GRU and LSTM each, followed by the dropout with a rate of 0.5. After flattening, the layer is the output layer with the softmax function. Other hyperparameters are the same as other networks for better comparison.

### 3.5. Corresponding Responses

After the sentiment analysis processing, LUX has a proper response to each sentiment. There are three prepared categories to choose from: playing music, reciting wise sayings, and speaking sympathizing words. For decades, music therapy tries to release depression and negative emotions. Thus, providing music by corresponding response is suited. Some researchers have found music beneficial in decreasing stress and depression [32,33]. We get commonly spoken wise sayings to provide users with emotional relief. Furthermore, LUX also speaks sympathizing words to users as a form of digital therapy. The corresponding task takes advantage of the fact that smart mirrors can familiarize the user.

One of these three methods is chosen as a response to the user’s sentiment. The category “sympathizing” would not apply to the label “happiness”. Table 2 lists the possible responses corresponding to each sentiment.

#### Recommender System

**Figure 4:**
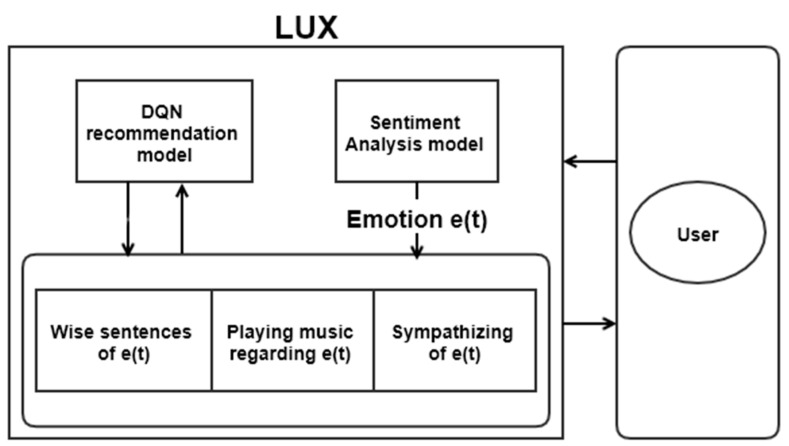
DQN algorithm for recommending user response.

We implemented reinforcement learning (RL) as a recommender system for a user’s emotion and the actions that correspond to it. We randomly choose the three methods; however, the recommender system algorithm would provide more precise results to secure the experimental user group. We proposed a recommender system based on a deep Q network (DQN) to select the proper response. DQN is adequately used in the recommender system to recommend better sources to users and evaluate the algorithm according to the feedback [34]. State S would be the emotional state of the user, as given by Algorithm 1. Action A is the set of responses in Table 2—the discount factor as γ, and the epsilon for exploring as ϵ. The discount factor is set to 1, so that past results affect the existing sentence without exception, while the existing user response does not fade away. We defined the reward as r and the replay buffer as R. The proposed DQN model is Q, and the target model of $Q\;is\;Q^{\prime }$. If the user likes a given response, they can provide a binary reward. Through the model, LUX can select the proper response to the user. The proposed method is summarized in Algorithm 1 and Figure 4.
**Algorithm 1** DQN algorithm to recommend user responses after sentiment analysis.1: **Input**: Initialize state S, action A, discount factor γ=1, epsilon ϵ, reward r, and replay buffer R. 2: **Output**: New recommendation 3: Initialization weight W with kaiming uniform 4: DQN Model Q, target Model $Q^{\prime }$ 5: sentence t 6: **While**: 7: State S(t)← emotion probability of the user 8: Action A(t)← Q(S(t)) with probability 1−ϵ, otherwise explore the given actions 9: r(t)← User responding with ‚good ‘and ‚bad ‘according to action. 10: Save {S(t), A(t), r(t)} in R 11: Train Q and update $Q^{\prime }$to Q(t) ← r(t)+γ∗max(Q(t+1)) 12: t← user sentence 13: **if** the user does not say any more: 14: **break** 15: **End**

## 4. Implementation of Smart Mirror LUX

**Figure 5:**
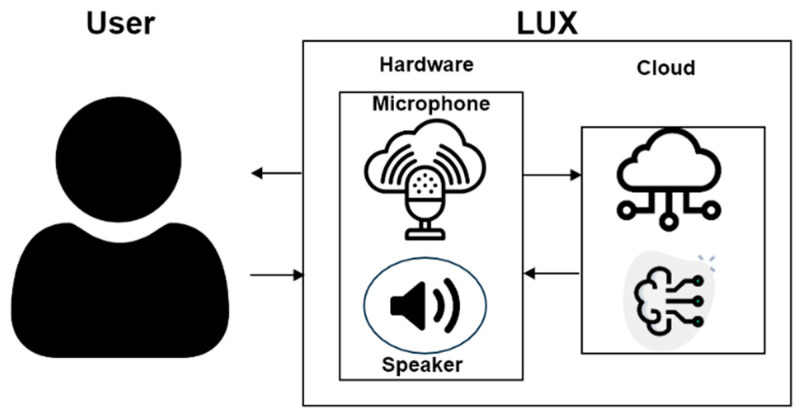
Interaction between LUX and the user.

The previous section elaborated upon the implementation of the sentiment analysis algorithm for LUX. LUX consummates by the software, mirror display UI, mirror frame, and basic hardware, containing speaker and microphone module. Figure 5 describes the interaction between a user and LUX. When the user speaks, the speaker module detects, and STT API extracts voice to text. After that, the wake-up prompt and sentiment analysis proceed, as explained in Section 3. During the task, the speaker module delivers responses to the user. The socket transmission is used to connect the hardware and the server.

**Figure 6:**
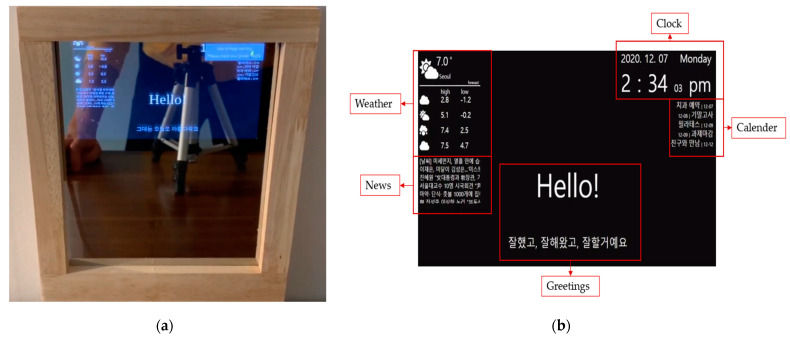
The smart mirror LUX: (**a**) the front view model of LUX, and (**b**) the display view of LUX, including weather, clock, calendar, and news.

**Figure 7:**
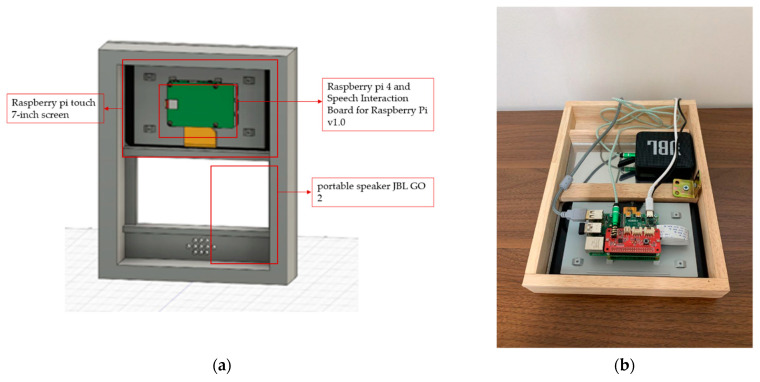
LUX hardware: (**a**) hardware as a half-mirror shape; (**b**) back view of the LUX.

LUX consists of a Raspberry Pi Touch 7-inch screen, Raspberry Pi 4, portable speaker JBL GO 2, Speech Interaction Board for Raspberry Pi, and an auxiliary battery. The front case is an invisible acrylic attached to a half mirror film that functions as a mirror. As an advantage of a smart mirror, we can implement a beautiful design using woods connected with horizontal brackets. Similar to existing smart mirrors, our display presents the user’s location’s calendar and weather, as shown in Figure 6. The front of LUX is described in Figure 6a. The edge of the mirror is made of wood, and if the user turns it off, it also functions as a standard mirror. The graphic user interface of LUX that displays weather, a clock, a calendar with the user’s schedule, and news, similar to what existing smart mirrors provide, are shown in Figure 6b. The speaker module inside LUX can be activated by a wake-up prompt. The concept of design and overside of the hardware is described in Figure 7a. It shows the space for the Raspberry Pi Touch 7-inch screen, battery, speaker, and microphone modules. The back view of the hardware, shown in Figure 7b, includes all the internal components.

As stated in the introduction, the smart mirror is a suitable device for interactive sentiment analysis. Like a mirror that shows external beauty, the smart mirror LUX can return an emotionally sensitive answer to show and handle internal sentiment.

## 5. Experimental Results

### 5.1. Test Datasets

We use 20% of the datasets for evaluating the sentiment analysis. The combined dataset consists of the AI Hub dataset and an Instagram-crawling dataset. The test dataset contained a total of 4766 sentences in four classes: anger (1263), sadness (1235), neutral (940), and happiness (1328).

### 5.2. Test Evaluation

We chose the *F*1 score, precision, recall, and accuracy for performance evaluation. Recall is calculated as

(1) $$Recall=\frac{TP}{TP+FN}\;$$

where *TP* denotes true positives, and *FN* denotes false negatives. Precision is obtained using the equation

(2) $$Precision=\frac{TP}{TP+FP}\;$$

where *FP* denotes false positives. The *F*1 score, which is the harmonic mean of performance Evaluations 1 and 2, can be calculated as

(3) $$F1-score=2*\frac{Precision\;*\;Recall}{Precision+Recall\;}.$$

**Figure 8:**
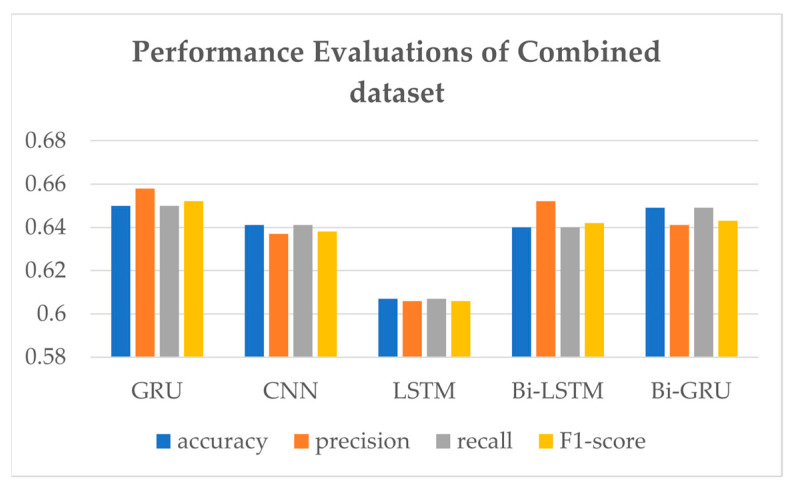
Bar table of performance evaluations of the combined dataset.

A comparison of the performance evaluations of various models with combined datasets is presented in Figure 8 and Table 3, rounded to the fourth decimal place. In the experiment result, the accuracy of SVM is so low (as low as 0.4) that its result is not included in the comparison results. Deep learning models such as LSTM [23], CNN [25], Bi-LSTM, Bi-GRU, and GRU are compared for sentiment analysis of Korean sentences. It shows that GRU shows the highest performances in the combined dataset. Also, since the bidirectional models’ training time was five times higher than other models, we selected GRU network as a deep learning sentiment analysis model for LUX. GRU presents an f1-score of 0.652, which is the highest. Table 3 also shows GRU accuracy and recall of 0.650 and precision of 0.658, which are the highest. LSTM shows the lowest performance evaluation among the models.

**Figure 9:**
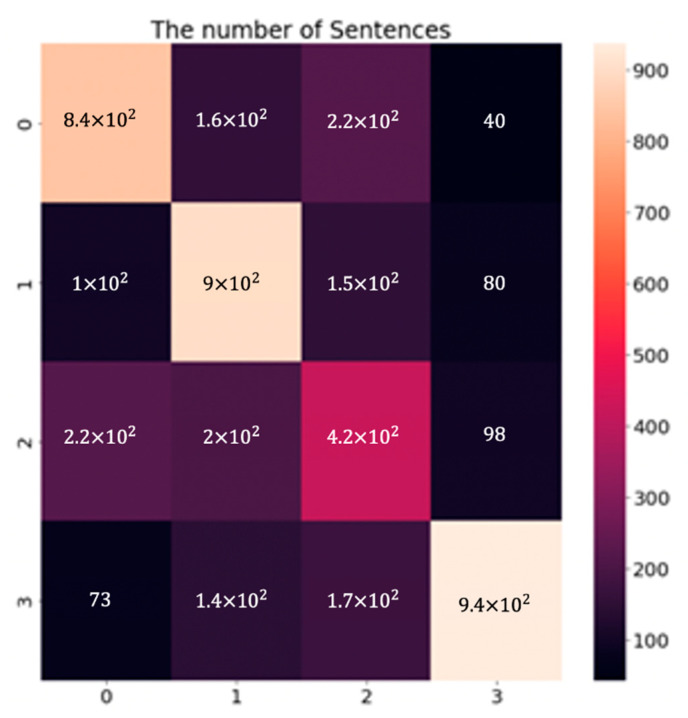
The confusion matrix of the GRU network evaluation result combined datasets with four labels, 0 (Anger), 1 (Sadness), 2 (Neutral), and 3 (Happiness).

The performance evaluations of the GRU model to compare each label are shown in Table 4. The confusion matrix of the GRU model with the combined dataset is shown in Figure 9. Data labels match the numbers 0, 1, 2, and 3 to anger, sadness, neutral, and happiness, respectively. The numbers describe the numbers of the data. We can confirm that the last label, “happiness” demonstrates outstanding performance. As shown in Table 4, the remaining results are 0.681, 0.642, 0.434, and 0.809 for precision, 0.667, 0.730, 0.447, and 0.705 for recall, and 0.674, 0.683, 0.440, and 0.753 for the F1-score of each category. We guessed that the lower performance evaluation of the “neutral” label is due to its inherent vagueness in terms of classification.

**Figure 10:**
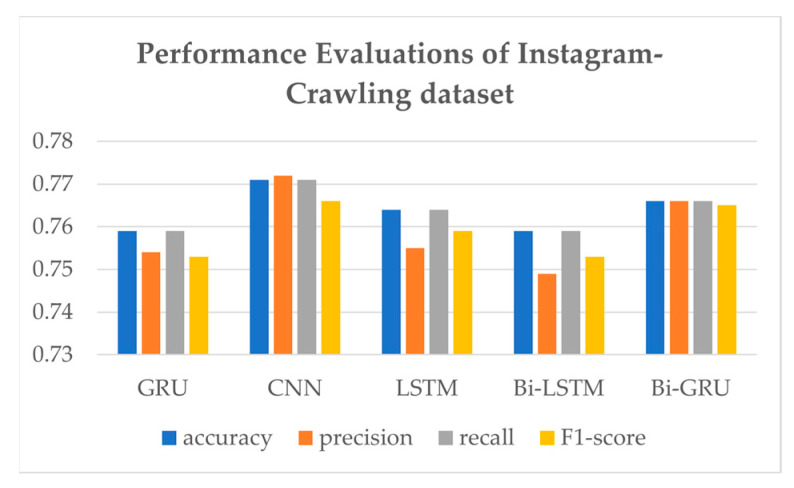
Bar table of evaluation performances of models.

As LUX has a primary purpose of handling everyday conversations, further experiments using only Instagram-crawling dataset experiments are shown in Table 5 and Figure 10. Interestingly, CNN shows the highest performance evaluation, with an accuracy of 0.771, precision of 0.772, recall of 0.771, and F1-score of 0.766.

To figure out if sentences translated to English may lead to better results, we conducted an experiment with the Korean-to-English translator. The randomly sampled 846 sentences of the AI Hub dataset were applied after translation using NAVER open API. GRU, CNN, and embeddings from language model (ELMo) models were used to compare results. Table 6 shows that GRU performs at an accuracy of 0.441, the accuracy of CNN is 0.406, and ELMo is 0.518, which is the highest but is not better than the performance with the original Korean dataset.

## 6. Conclusions

This paper proposed a new smart mirror, LUX, that provides sentiment analysis of human speech, supporting a more comfortable lifestyle to complement the exhausting reality. The sentiment analysis model of LUX describes the possibility of caring for more accurate emotional states with four labels: anger, sadness, neutral, and happiness. The case study showed that the highest performance evaluation of Korean text sentiment analysis for the combined AI hub and Instagram-crawling dataset is the accuracy of 0.650, the precision of 0.658, recall of 0.650, and F1-score of 0.652 by using GRU. Higher accuracy can be achieved with a larger dataset. The experiment of the Instagram-crawling dataset shows that everyday conversation sentences would prefer CNN. If more everyday conversation datasets would be gathered, we could have further studies on finding the optimal model. The experiment also suggested the ease of classifying the “happiness” label above the others; the maximum F1-score for “happiness” was 0.809, which will be a good insight for subsequent research. This work focuses on building supplements for maintaining mental health with the proper device. LUX’s main contribution is sentiment analysis in smart mirror architecture with good performance and three possible interactive responses: reciting wise sayings, playing music, and sympathizing. In light of the current COVID-19 crisis, the study of LUX, which supports a healthy mental state, is highly significant.

Future work will improve the accuracy of sentiment analysis for NLP tasks for text and add voice intonation to support the analysis of user emotions. The proposed DQN model for recommending the proper response according to the emotion will be implemented. The recommender system will eventually be able to provide interactive and customized digital therapy.

## Figures and Tables

**Table 1:** Dataset distribution of Instagram-crawling.

Emotions	Keywords	The Number of Keyword Data	The Number of Emotion Data
Anger	화가 난다 (I am angry)	302	535
	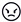	156	
	ETC	77	
Sadness	에휴 (Sigh)	24	808
	지친다 (I am exhausted)	181	
	아프다 (I am sick)	214	
	ETC	389	
Neutral	ETC	31	31
Happiness	행복해 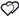 (I am happy)	400	656
	기분 좋은 날 (It’s a good day)	166	
	ETC	90	

**Table 2:** Responses corresponding to each of the labels.

	Wise Saying	Playing Music	Sympathizing
Labels	Anger	Anger	Anger
	Sadness	Sadness	Sadness
	Neutral	Neutral	Neutral
	Happiness	Happiness	

**Table 3:** Performance comparison of various models trained with combined dataset.

Models	Accuracy	Precision	Recall	F1-Score
GRU	0.650	0.658	0.650	0.652
CNN	0.641	0.637	0.641	0.638
LSTM	0.607	0.606	0.607	0.606
Bi-LSTM	0.640	0.652	0.640	0.642
Bi-GRU	0.649	0.641	0.649	0.643

**Table 4:** Precision, Recall, and F1-score of GRU model trained by the combined dataset.

	Precision	Recall	F1-Score
Anger	0.681	0.667	0.674
Sadness	0.642	0.730	0.683
Neutral	0.434	0.447	0.440
Happiness	0.809	0.705	0.753

**Table 5:** Performance comparison of various models trained by the Instagram-crawling dataset.

Models	Accuracy	Precision	Recall	F1-Score
GRU	0.759	0.754	0.759	0.753
CNN	0.771	0.772	0.771	0.766
LSTM	0.764	0.755	0.764	0.759
Bi-LSTM	0.759	0.749	0.759	0.753
Bi-GRU	0.766	0.766	0.766	0.765

**Table 6:** Accuracy when translating AI hub dataset to English.

	GRU	CNN	ELMo
Accuracy	0.441	0.406	0.518

## Data Availability

Not applicable.

## References

[1] Soleymani M., Garcia D., Jou B., Schuller B., Chang S.-F., Pantic M. (2017). A survey of multimodal sentiment analysis. Image Vis. Comput..

[2] Zhang Y., Tiwari P., Song D., Mao X., Wang P., Li X., Pandey H.M. (2021). Learning interaction dynamics with an interactive LSTM for conversational sentiment analysis. Neural Netw..

[3] Kouloumpis E., Wilson T., Moore J. Twitter sentiment analysis: The good the bad and the omg! In Proceedings of the International AAAI Conference on Web and Social Media, Catalonia, Spain, 17–21 July 2011.

[4] Denecke K., Deng Y. (2015). Sentiment analysis in medical settings: New opportunities and challenges. Artif. Intell. Med..

[5] Jang Y.-B. (2019). Effects of AI Speaker Users’ Usage Motivations and Perception of Relationship Type with AI Speaker on Enjoyment. J. Korea Contents Assoc..

[6] Purington A., Taft J.G., Sannon S., Bazarova N.N., Taylor S.H. “Alexa is my new BFF” Social Roles, User Satisfaction, and Personification of the Amazon Echo. Proceedings of the 2017 CHI Conference Extended Abstracts on Human Factors in Computing Systems.

[7] Smart Mirror Market–Growth, Trends, COVID-19 Impact, and Forecasts (2021–2026). https://www.mordorintelligence.com/industry-reports/smart-mirror-market.

[8] Rajcic N., McCormack J. Mirror Ritual: An Affective Interface for Emotional Self-Reflection. Proceedings of the 2020 CHI Conference on Human Factors in Computing Systems.

[9] D’Souza A.A., Kaul P., Paul E., Dhuri M. (2019). Ambient Intelligence Using Smart Mirror-Personalized Smart Mirror for Home Use. Proceedings of the 2019 IEEE Bombay Section Signature Conference (IBSSC).

[10] Garcia I.C.A., Salmon E.R.L., Riega R.V., Padilla A.B. (2017). Implementation and Customization of a Smart Mirror through a Facial Recognition Authentication and a Personalized News Recommendation Algorithm. Proceedings of the 2017 13th International Conference on Signal-Image Technology & Internet-Based Systems (SITIS).

[11] Athira S., Francis F., Raphel R., Sachin N., Porinchu S., Francis S. Smart mirror: A novel framework for interactive display. Proceedings of the 2016 International Conference on Circuit, Power and Computing Technologies (ICCPCT).

[12] Besserer D., Bäurle J., Nikic A., Honold F., Schüssel F., Weber M. Fitmirror: A smart mirror for positive affect in everyday user morning routines. Proceedings of the Workshop on Multimodal Analyses Enabling Artificial Agents in Human-Machine Interaction.

[13] Njaka A.C., Li N., Li L. (2018). Voice Controlled Smart Mirror with Multifactor Authentication. Proceedings of the 2018 IEEE International Smart Cities Conference (ISC2).

[14] Cvetkoska B., Marina N., Bogatinoska D.C., Mitreski Z. Smart mirror E-health assistant—Posture analyze algorithm proposed model for upright posture. Proceedings of the IEEE EUROCON 2017-17th International Conference on Smart Technologies.

[15] Trilla A., Alias F. (2012). Sentence-Based Sentiment Analysis for Expressive Text-to-Speech. IEEE Trans. Audio Speech Lang. Process..

[16] Shaver P., Schwartz J., Kirson D., O’connor C. (1987). Emotion knowledge: Further exploration of a prototype approach. J. Pers. Soc. Psychol..

[17] AI Hub. https://aihub.or.kr/.

[18] Hochreiter S., Schmidhuber J. (1997). Long short-term memory. Neural Comput..

[19] An J., Kim H.-W. (2015). Building a Korean Sentiment Lexicon Using Collective Intelligence. J. Intell. Inf. Syst..

[20] Park S.J., Lim Y.S., Sams S., Nam S.M., Park H.W. (2011). Networked politics on Cyworld: The text and sentiment of Korean political profiles. Soc. Sci. Comput. Rev..

[21] Jang H., Shin H. Effective use of linguistic features for sentiment analysis of korean. Proceedings of the 24th Pacific Asia Conference on Language, Information and Computation.

[22] Jung Y., Park K., Lee T., Chae J., Jung S. (2017). A corpus-based approach to classifying emotions using Korean linguistic features. Clust. Comput..

[23] Song M., Park H., Shin K.-S. (2019). Attention-based long short-term memory network using sentiment lexicon embedding for aspect-level sentiment analysis in Korean. Inf. Process. Manag..

[24] Do H.J., Choi H.-J. Korean twitter emotion classification using automatically built emotion lexicons and fine-grained features. Proceedings of the 29th Pacific Asia Conference on Language, Information and Computation: Posters.

[25] Park H.-J., Song M.-C., Shin K.-S. (2018). Sentiment Analysis of Korean Reviews Using CNN: Focusing on Morpheme Embedding. J. Intell. Inf. Syst..

[26] Dashtipour K., Gogate M., Li J., Jiang F., Kong B., Hussain A. (2020). A hybrid Persian sentiment analysis framework: Integrating dependency grammar based rules and deep neural networks. Neurocomputing.

[27] Oussous A., Benjelloun F.-Z., Lahcen A.A., Belfkih S. (2019). ASA: A framework for Arabic sentiment analysis. J. Inf. Sci..

[28] Chung J., Gulcehre C., Cho K., Bengio Y. (2014). Empirical evaluation of gated recurrent neural networks on sequence modeling. arXiv.

[29] Zhang L., Zhou Y., Duan X., Chen R. A hierarchical multi-input and output bi-GRU model for sentiment analysis on customer reviews. Proceedings of the IOP Conference Series: Materials Science and Engineering.

[30] Agarwal A., Xie B., Vovsha I., Rambow O., Passonneau R.J. Sentiment analysis of twitter data. Proceedings of the Workshop on Language in Social Media (LSM 2011).

[31] Park E.L., Cho S. KoNLPy: Korean natural language processing in Python. Proceedings of the Annual Conference on Human and Language Technology.

[32] Bak H., Lee J. (2014). A meta-analysis of the music therapy research to reduce stress. Korean J. Music. Ther..

[33] Moon C.B., Lee J.Y., Kim D.-S., Kim B.M. (2019). Analysis of Mood Tags for Music Recommendation. J. Korea Ind. Inf. Syst. Res..

[34] Zhao X., Zhang L., Ding Z., Xia L., Tang J., Yin D. Recommendations with negative feedback via pairwise deep reinforcement learning. Proceedings of the 24th ACM SIGKDD International Conference on Knowledge Discovery & Data Mining.

